# Validation of the eHealth Literacy Scale Instrument in a Restless Legs Syndrome Population: Classical Test Theory and Rasch Analysis Study

**DOI:** 10.2196/68474

**Published:** 2025-09-10

**Authors:** Mattias Georgsson, Elzana Odzakovic, Maria Björk, Viktor Kaldo, Susanna Jernelöv, Kerstin Blom, Martin Ulander, Bengt Fridlund, Susanne Knutsson, Christina Sandlund, Amir Pakpour, Anders Broström

**Affiliations:** 1 Department of Nursing, School of Health and Welfare, Jönköping University, Gjuterigatan 5, Jönköping, 553 18, Sweden, 46 036101000; 2 Department of Psychology, Faculty of Health and Life Sciences, Linnaeus University, Växjö, Sweden; 3 Centre for Psychiatry Research, Department of Clinical Neuroscience, Karolinska Institutet, & Stockholm Health Care Services, Region Stockholm, Stockholm, Sweden; 4 Division of Psychology, Department of Clinical Neuroscience, Karolinska Institutet, Stockholm, Sweden; 5 Division of Neurobiology, Department of Biomedical and Clinical Sciences, Linköping University, Linköping, Sweden; 6 Department of Clinical Neurophysiology, Linköping University Hospital, Linköping, Sweden; 7 Centre of Interprofessional Collaboration within Emergency Care (CICE), Linnaeus University, Växjö, Sweden; 8 Department of Health and Caring Sciences, Faculty of Health and Life Sciences, Linnaeus University, Växjö, Sweden; 9 Department of Neurobiology, Care Sciences and Society, Karolinska Institutet, Stockholm, Sweden; 10 Academic Primary Health Care Centre, Region Stockholm, Stockholm, Sweden; 11 Department of Health and Caring Sciences, Western Norway University of Applied Sciences, Bergen, Norway

**Keywords:** restless legs syndrome, health literacy, decisional conflict, shared decision-making, sleep, confirmatory factor analysis, validity

## Abstract

**Background:**

An increased use of the internet and digital health care for patients with long-term conditions implies a need for assuring digital health literacy skills. Patients with restless legs syndrome (RLS) represent a group where digital sources of information are highly valued. This is due to a difficult diagnosis and complex treatment situation that contributes to patients seeking out digital resources themselves to handle the perceived shortcomings in their care. To benefit from these resources, patients need to have the digital skills to explore information to optimize their understanding of the disease and its treatments. The eHealth Literacy Scale (eHEALS), which has been used in both general populations and patients with long-term conditions, could, if proven valid, be used by researchers and clinicians to assess digital health literacy among patients with RLS to inform the development of patient-centered digital health care information and interventions.

**Objective:**

The aim of the study is to investigate the psychometric properties of eHEALS in patients with RLS to determine its adequacy and potential utility.

**Methods:**

A cross-sectional design including patients with RLS from the Swedish national RLS patient organization was used. Data were collected via a mail-based survey comprising back-and-forth translated Swedish versions of the following instruments: eHEALS, Restless Legs Syndrome-6 Scale (RLS symptoms), Pittsburgh Sleep Quality Inventory (sleep quality), Epworth Sleepiness Scale (daytime sleepiness), Patient Health Questionnaire-9 (depressive symptoms), and CollaboRATE (shared decision-making). Confirmatory factor analysis and Rasch models were used to assess the validity and reliability of the eHEALS. Measurement invariance, unidimensionality, and differential item functioning across age, gender, medication use, sleep quality, level of depressive symptoms, and participation in care decisions were assessed.

**Results:**

A total of 788 patients with a mean age of 70.8 (SD 11.3) years participated. Among them, 64.7% (n=510) were women, 73.8% (n=582) were married or living together, and 43.5% (n=343) had attained a university education. A median eHEALS score of 28 (IQR 22-33) was reported. The unidimensionality of the eHEALS was supported by the confirmatory factor analysis and the Rasch model. The reliability of the eHEALS was confirmed using composite reliability and Cronbach α. No differential item functioning was identified for age, gender, medication use, shared decision-making condition, depressive symptoms, or sleep quality, meaning that these groups do not have different probabilities of endorsing a given item after controlling for the overall score.

**Conclusions:**

The eHEALS showed good validity and reliability and operated equivalently for men and women of different ages with various clinical and treatment conditions related to RLS. Accordingly, health care professionals can use eHEALS as a psychometrically sound tool to explore the digital health literacy level among patients with RLS.

## Introduction

Today, there is an increased reliance on the internet and digital health care. About 5.4 billion (67%) people in the world are internet users [1], and in a highly IT-dependent country like Sweden, this number is currently 95% of the population [2]. In its Global Strategy on Digital Health 2020‐2025, the World Health Organization [3] stresses the increased importance of digital health and to deliver it with quality. The increasing proportion of older people with long-term conditions requires frequent care contacts, such as restless legs syndrome (RLS) [4]. This means that understanding the individuals’ ability to obtain specific knowledge about their disease diagnosis and its treatment via digital information channels becomes more important. The eHealth Literacy Scale (eHEALS) [5] is an instrument used to evaluate competence in health information–seeking behavior both in the general population as well as in patients with specific diagnoses. The eHEALS can be suitable for providing guidance to health care professionals regarding prerequisites for information-seeking behavior [6] and digitally delivered health care interventions [7] but has not yet been psychometrically evaluated in patients with RLS.

RLS is a chronic disorder [4] where the patient has an irresistible need to move their legs and commonly experiences poor sleep [8-10] and depression [11], with a significant impact on the whole life situation [12-14]. Moreover, decreased physical function, lower general health, vitality, and quality of life (QoL) are described in comparison to the general population [15]. According to a recent meta-analysis, RLS has a prevalence of 3% in the general population, and it is more common among women and older people [16]. Some patients describe a prolonged period of symptoms before a diagnosis is made [13], which might be related to varied descriptions of symptoms [17,18]. A multiple pharmacological treatment strategy, including iron, α2δ channel ligands, and benzodiazepines [19], is often used together with dopaminergic drugs. Nonpharmacological therapies, including self-care can also be applied, but evidence of their effectiveness is lacking [20-22]. These diagnostic and treatment-related difficulties [23,24] likely contribute to an increased desire among patients to seek RLS-related information from digital sources. Those with skills to identify relevant information (ie, good health literacy) might improve their knowledge and insight before and after health care visits [13], which most likely can affect the view of patients with RLS on shared decision-making of treatment [25] and in the long run treatment adherence.

eHealth literacy is “the ability to seek, find, understand, and appraise health information from electronic sources and apply the knowledge gained to addressing or solving a particular health concern” [5]. Shiferaw et al [7] found both internet use and eHealth literacy levels in patients with long-term conditions to be low and highlighted the importance of attending to this deficiency. Interestingly, internet-delivered cognitive behavioral therapy (ICBT) interventions have become popular since they can improve functioning in patients with conditions, such as chronic pain [26], asthma [27], and atrial fibrillation [28], where they also improved QoL [29]. Face-to-face–delivered cognitive behavioral therapy has, in one recent RLS study [30], been found to decrease insomnia symptoms. Since cognitive behavioral therapy for insomnia is proven highly effective when delivered via the internet, this suggests ICBT as a potential complement to traditional pharmacological RLS treatment. However, if older patients with RLS are to use digital sources to either seek information about their disease condition or participate in ICBT, it is vital that they possess health literacy and, in particular, eHealth literacy skills to ascertain the use of these resources in an efficient and purposeful manner. One initial step could be to consider the eHealth literacy level among patients with RLS with the use of the eHEALS [5].

The eHEALS has been used in patients with long-term conditions such as cancer [31-34] and rheumatic conditions [35]. It has also been validated in general populations in Italian [36] and Portuguese [37], as well as in targeted cardiovascular disease populations in Persian [38], Norwegian [39,40], and German [41]. However, despite having been validated and proven useful in the earlier-mentioned general populations, as well as in various disease populations, eHEALS may not be applicable in patients with RLS, who are predominantly older people, as no studies have explored its psychometric properties in this patient group. More specifically, it may be important to investigate subgroups of patients with RLS. This entails those patients who report severe RLS symptoms, sleep disturbances, and depressive symptoms, which have been found to affect cognitive ability [8], as those who experience these symptoms may have a different likelihood to identify and understand health information from, for example, electronic sources [2]. Patients with RLS, who have varied symptoms and where effective treatment is not always available or effective [24], often turn on the web for knowledge about their disease [13]. If future dependency on digital health resources continues to increase, demanding adequate skills and abilities among users [2], knowledge about eHealth literacy measured with valid instruments for older patients with RLS will likely be important. As eHEALS is the most frequently used instrument to assess digital health literacy level and determine a patient’s digital health care engagement [42], it warrants further investigation for this purpose in this specific patient group. The aim of this study was to investigate the psychometric properties of eHEALS in patients with RLS to determine its adequacy and potential utility.

## Methods

### Study Design and Participants

A cross-sectional design was used, including patients with RLS recruited from the Swedish RLS Association, a nationwide patient organization with 1500 members. Inclusion criteria for filling out the cross-sectionally administered postal survey were as follows: (1) being 18 years or older of age, (2) diagnosed and treated for RLS, (3) able to speak and understand Swedish, and (4) provide a written informed consent.

### Data Collection

Information about the aim of the study was sent to the Swedish RLS Association Board, which allowed it to be shared with their listed members. To participate, eligible members had to return a written informed consent form and the completed survey in a prestamped envelope. Information provided by the participants also included their age, gender, employment, economic situation, as well as years since their RLS diagnosis, self-reported comorbidities, and treatment.

### Instruments

Back-and-forth translated Swedish versions of eHEALS, Restless Legs Syndrome-6 Scale (RLS-6), Pittsburgh Sleep Quality Index (PSQI), Epworth Sleepiness Scale (ESS), Patient Health Questionnaire-9 (PHQ-9), and CollaboRATE were used to collect data.

#### The eHealth Literacy Scale

The eHEALS is a self-administered instrument that includes 8 items scored on a 5-point Likert-type scale and was used to assess the level of eHealth literacy [5]. The 8 items determine the patient’s ability to seek, find, evaluate, and use digital information for decisions regarding their health. A Likert score of 1=strongly disagree and 5=strongly agree. Scores range from 8 to 40, and the higher the score, the higher the level of the patient’s eHealth literacy [5]. When tested in a general Swedish population, eHEALS was assessed as being unidimensional with high internal consistency [43].

#### Restless Legs Syndrome-6 Scale

The well-validated RLS-6 was used to determine the severity of daytime and nighttime RLS symptoms [44,45]. The 6 items involve sleep quality, RLS experiences during night and daytime, as well as during activity to differentiate RLS from other disorders (left out in scoring). Items are scored on a 0‐10 scale, with 0=no symptoms and 10=very severe symptoms [44]. The RLS-6 has been used in previous Swedish RLS studies [14,25].

#### The Pittsburgh Sleep Quality Index

The well-established PSQI was used to assess sleep quality and sleep disturbances during the last month [46]. It includes 7 components with items involving a wide range of applicable indicators for evaluating sleep quality, sleep latency, sleep duration, sleep efficiency, sleep disturbances, use of sleep medications, and daytime dysfunction. To calculate PSQI global scores, the 7 components are each rated from 0 to 3 points, stemming in a global score that ranges from 0 to 21 points. A score of ≥5 implies sleep difficulties [46]. The PSQI has proven valid and reliable in patients with primary insomnia [47] and multiple sclerosis [48]. PSQI can be used to distinguish sleep disorders [49,50].

#### The Epworth Sleepiness Scale

The well-established ESS was used to determine the degree of daytime sleepiness [51]. It includes 8 different situations, all scored on a scale of 0‐3, in which the patient assesses the risk of dozing off or falling asleep. The total score ranges between 0 and 24, and a score >11 indicates excessive daytime sleepiness [51,52].

#### Patient Health Questionnaire-9

The well-validated PHQ-9, a 9-item questionnaire, was used to determine depressive symptom severity [53-55]. Each item is scored from 0=not at all to 3=nearly every day. The cutoff points that determine the level of severity ranging from mild to severe depressive symptoms are 5, 10, 15, and 20. The PHQ-9 score can range from 0 to 27 [53].

#### CollaboRATE

The CollaboRATE, a 3-item instrument, was used to measure shared decision-making [56]. The first item measures the effort made to help the patient to understand his or her health issues; the second item measures the effort made to listen to what matters most to the patient about his or her health issues, and the third item concerns the effort made to include the patient in his or her future care. Each item is scored on a 5-point Likert scale, where 1 signifies no effort was made and 5 that every effort was made [57]. CollaboRATE has shown good validity and reliability in Swedish patients with RLS [25].

### Statistical Analysis

#### Internal Consistency

Internal consistency was tested by computing Cronbach α and McDonald ω as well as composite reliability. Coefficients with values >0.70 indicated an acceptable level [58,59]. The internal consistency of the eHEALS was further assessed by calculating item-total correlation (corrected for overlap). The correlation coefficients ≥0.4 were considered acceptable.

#### Convergent and Discriminant Validity

To evaluate convergent and discriminant validity, Pearson correlation coefficients were calculated between eHEALS scores and related constructs, including daytime sleepiness (ESS), RLS symptom severity (RLS-6), sleep quality (PSQI), depressive symptoms (PHQ-9), and shared decision-making (CollaboRATE).

#### Construct Validity

Construct validity of the eHEALS was tested using confirmatory factor analysis (CFA) [60]. Considering the nature of ordinal data with Likert response options (from 1 to 5), the diagonally weighted least squares estimation method was used in the CFA. Several model fit indices were used to evaluate the unidimensional structure in the CFA model: the chi-square (*χ*^2^) and degrees of freedom (*df*), the root mean square error of approximation (RMSEA), Tucker-Lewis index (TLI), Comparative Fit Index (CFI), and standardized root mean square residual (SRMR). An acceptable CFA model fit has a CFI, TLI value of 0.95 or higher, an RMSEA and SRMR value of 0.08 or lower, and a chi-square to degrees of freedom ratio (*χ*^2^/*df*) lower than 5 [61]. The average variance extracted was calculated to assess the convergent validity of the eHEALS.

To make sure that the association between the latent structure of electronic health literacy and its 8 items was equal across subgroups of patients (ie, age and gender), measurement invariance was used. A series of multiple groups of CFAs was conducted on the data to explore measurement invariance across the 2 given subgroups of patients [60]. A hierarchical approach to measurement invariance was considered: at the first level (the lowest restrictive model), configural invariance was examined, assessing whether the pattern of relationships between the eHEALS items and the factor was consistent across the groups. In the next level, metric invariance was conducted to determine whether the factor loadings were equal across the groups. In the last level (the highest restrictive level), scalar invariance was conducted to assess whether the item intercepts were equal across the groups. Measurement invariance was established if the differences between the hierarchical models were nonsignificant, as indicated by a nonsignificance of chi-square difference, ∆CFI<0.01, ∆RMSEA<0.03, and ∆SRMR<0.01 [62].

The psychometric properties of the eHEALS were further examined using Rasch analysis with the rating scale model. To test item fit in the Rasch model, infit and outfit mean squares (MNSQs) were used, with values between 0.7 and 1.3 indicating an acceptable range [63]. To ensure that various subgroups of patients (ie, age, gender, medication use, shared decision-making condition [CollaboRATE], depressive symptoms [PHQ-9], and sleep quality [PSQI]) interpreted the items in eHEALS similarly, differential item functioning (DIF) was conducted. A contrast >0.5 logit was considered substantial [64].

To compare participants with distinctly different levels of eHealth literacy, the total eHEALS scores were divided into 2 groups using a median split. Participants scoring 28 or below were classified as having low eHealth literacy, and those scoring above 28 were categorized as having high eHealth literacy.

All statistical analyses were performed using the SPSS (version 28; IBM Corp), Winsteps software (version 4.3.0; Institute for Objective Measurement, Inc), and Jeffreys’ Amazing Statistical Program (version 0.18.03.0; JASP Team).

### Ethical Considerations

The study received ethics approval from the Swedish Ethical Review Authority (Dnr 2022-01515-01). All procedures and administration of data were conducted in line with the General Data Protection Regulation and ethical principles outlined in the Helsinki Declaration [65]. Informed written consent was obtained from all study participants regarding data collection and the analysis of the data. The survey was submitted anonymously by participants, and no compensation was provided.

## Results

### Study Population

A total of 788 patients with a mean age of 70.8 (SD 11.3; range 28‐94) years responded (ie, response rate of 52%). Among them, 64.7% (n=510) were women, 73.8% (n=582) were married or living together, and 43.5% (n=343) had attained a university education. Additionally, 72.9% (n=575) were retired. The mean time since being diagnosed with RLS was 16.4 (SD 10.5) years. Of comorbidities, hypertension (n=281, 35.6%) and cardiovascular disease (n=143, 18.1%) were the most common. Iron deficiency was reported by 9.8% (n=78), and 3.6% (n=29) reported severe depressive symptoms. Dopamine agonists were the most common drugs used by 79.3% (n=625) of the patients. Satisfaction with prescribed RLS treatment was reported by one-fifth. Regarding RLS symptom severity, 44% (n=347) and 37% (n=292) reported severe symptoms during night- and daytime, respectively, based on the RLS-6 questionnaire. A total of 43% (n=339) experienced excessive daytime sleepiness. A median eHEALS score of 28 (IQR 22‐33) was reported. Table 1 presents patient demographics and clinical characteristics.

**Table 1.** Sociodemographic characteristics of the population.

Variables	Values (N=788)
Gender, n (%)	
Women	510 (64.7)
Age (years)	
Mean (SD)	70.8 (11.3)
Range	28‐94
Civil status, n (%)	
Married or living together	582 (73.8)
Educational level, n (%)	
9 years or below	156 (19.8)
12‐13 years	267 (33.9)
University	343 (43.5)
Comorbidities, n (%)	
Migraine	59 (7.5)
Iron deficiency	78 (9.9)
Hypertension	281 (35.6)
Cardiovascular disease	143 (18.1)
Other	29 (3.6)
Pharmacological treatment, n (%)	
Dopamine agonists	625 (79.3)
Opioids	163 (20.6)
α_2_δ ligands	144 (18.2)
Dopa or derivatives	105 (13.3)
Iron supplement	33 (4.2)
Number of prescribed drugs, median (IQR)	3 (1-5)
RLS symptoms, median (IQR)	
RLS-6^b^ sleep quality	11 (8-14)
RLS-6 RLS at nighttime	9 (6-13)
RLS-6 daytime RLS manifestations during relaxation	4 (2-7)
Sleep, median (IQR)	
PSQI^c^ global score	12 (10-14)
Daytime sleepiness, n (%)	
ESS^d^>10 (excessive daytime sleepiness)	339 (43)
Depressive symptoms, median (IQR)	
PHQ-9^e^ total score	7 (4-11)
Shared decision-making, median (IQR)	
CollaboRATE total score	6 (3-9)
Competence in health information seeking, median (IQR)	
eHEALS^f^ total score	28 (22-33)

a RLS: restless legs syndrome.

b RLS-6: Restless Legs Syndrome-6 Scale.

c PSQI: Pittsburgh Sleep Quality Index.

d ESS: Epworth Sleepiness Scale.

e PHQ-9: Patient Health Questionnaire-9.

f eHEALS: eHealth Literacy Scale.

### Internal Consistency

The internal consistency, as measured by Cronbach α and McDonald ω and composite reliability, was above 0.95. Moreover, the item-total correlations (corrected for overlap) were all above 0.84 (Table 2).

**Table 2.** Psychometric properties of the eHealth Literacy Scale (eHEALS) at the scale level.

Psychometric testing	eHEALS
Internal consistency (Cronbach α)	0.968
Internal consistency (McDonald ω)	0.968
Confirmatory factor analysis	
*χ*^2^ (*df*)	52.6 (20)
Comparative Fit Index	0.997
Tucker-Lewis index	0.996
Root mean square error of approximation	0.047
Standardized root mean square residual	0.049
Average variance extracted	0.791
Composite reliability	
Item separation reliability from Rasch	0.98
Item separation index from Rasch	6.28
Person separation reliability from Rasch	0.92
Person separation index from Rasch	3.32

### Convergent and Discriminant Validity

Pearson correlation analyses showed that eHEALS scores were positively associated with shared decision-making as measured by the CollaboRATE total score (*r*=0.159; *P*<.001), indicating evidence of convergent validity. Weak but statistically significant negative correlations were found between eHEALS and depressive symptoms (PHQ-9; *r*=–0.09; *P*=.01) as well as daytime sleepiness (ESS; *r*=–0.081; *P*=.03), supporting aspects of discriminant validity. No significant correlations were observed between eHEALS and sleep quality (PSQI; *r*=–0.018; *P*=.63) or overall RLS symptom severity (*r*=–0.064; *P*=.08).

### Construct Validity

The factor structure of the eHEALS, examined by the CFA, is provided in Table 2. The unidimensional structure of the eHEALS showed a very good model fit with *χ*^2^_20_=52.5; *P*<.001; CFI=0.997; TLI=0.996; SRMR=0.049, except for RMSEA=0.047 (90% CI 0.031‐0.062). All factor loadings were significant and ranged from 0.856 (item 7) to 0.920 (item 3).

The unidimensional structure of eHEALS was then further examined to determine whether it could be interpreted similarly in different age and gender subgroups of the patients. As Table 3 shows, all items were perceived similarly by gender subgroups (a nonsignificant *χ*^2^ difference, ∆CFI<0.01, ∆RMSEA<0.03, and ∆SRMR<0.01). Although the *χ*^2^ difference test indicated a significant difference between the configural and metric models for age subgroups, the changes in CFI, RMSEA, and SRMR were all below the established thresholds for significance (ie, ∆CFI<0.01, ∆RMSEA<0.03, and ∆SRMR<0.01).

**Table 3.** Measurement invariance of the eHealth Literacy Scale across age and gender groups through confirmatory factor analysis.

Model and comparisons	Fit statistics							
	χ^2^ (*df*)	∆*χ*^2^ (∆*df*)	CFI^a^	∆CFI	SRMR^b^	∆SRMR	RMSEA^c^	∆RMSEA
Gender								
M1: configural	55.3 (40)	—^d^	0.998	—	0.051	—	0.032	—
M2: metric	60.9 (47)	—	0.999	—	0.053	—	0.028	—
M3: scalar	62.1 (54)	—	0.999	—	0.048	—	0.048	—
M2−M1	—	5.656 (7)	—	0.001	—	0.002	—	−0.004
M3−M2	—	1.146 (7)	—	0	—	−0.005	—	0.02
Age								
M1: configural	56.8 (40)	—	0.998	—	0.052	—	0.034	—
M2: metric	72.9 (47)	—	0.998	—	0.059	—	0.038	—
M3: scalar	76.8 (54)	—	0.998	—	0.054	—	0.034	—
M2−M1	—	16.114 (7)	—	0	—	0.007	—	0.004
M3−M2	—	3.917 (7)	—	0	—	−0.005	—	−0.004

a CFI: Comparative Fit Index.

b SRMR: standardized root mean square residual.

c RMSEA: root mean square error of approximation.

d Not applicable.

Item fit statistics from the Rasch model are presented in Table 2. All infit and outfit MNSQ values for the items were within the acceptable range: 0.75‐1.21 for infit MNSQ and 0.71‐1.26 for outfit MNSQ. Item 4 was reported to be the easiest item to interpret, while item 8 was perceived as the most difficult one (Table 4).

The results of the DIF analyses are presented in . No substantial DIF was found (ie, contrast >0.5) across age, gender, medication use, shared decision-making condition (CollaboRATE), depressive symptoms (PHQ-9), or sleep quality (PSQI). However, item 8 showed a potential DIF across sleep quality, indicating that those with sleep problems reported higher difficulty on item 8 compared to those without sleep problems (DIF=0.51).

Comparisons of patients’ characteristics between low and high health literacy groups are shown in Table 5. The results indicated that participants with higher literacy were significantly younger (mean age 68.1, SD 11.6 vs 73.1, SD 10.6 years; *P*<.001), more likely to be women (n=259, 70.2% vs n=225, 58.4%; *P*<.001), and more often had higher education levels (n=203, 56.4% vs n=126, 33.5%; *P*<.001). No significant differences were observed in civil status or comorbidity burden. Although RLS symptom severity, sleep quality (PSQI), and daytime sleepiness (ESS) were similar between groups, those with higher literacy reported significantly greater involvement in shared decision-making (CollaboRATE score: 6.89 vs 6.13; *P*=.001). A nonsignificant trend toward lower depressive symptoms (PHQ-9) was observed in the high literacy group (*P*=.05).

**Table 4.** Psychometric properties of the eHealth Literacy Scale (eHEALS) at the item level.

eHEALS	Factor loading^a^	Item-total correlation	Infit MNSQ^b^	Outfit MNSQ	Difficulty	Correlation	SE
Item 1	0.865	0.850	1.14	1.17	−0.05	0.88	0.07
Item 2	0.900	0.885	0.88	0.87	0.02	0.90	0.07
Item 3	0.920	0.906	0.75	0.71	−0.11	0.91	0.07
Item 4	0.884	0.869	1.15	1.09	−0.81	0.88	0.07
Item 5	0.917	0.901	0.78	0.79	−0.30	0.91	0.07
Item 6	0.901	0.882	0.93	0.93	0.05	0.90	0.07
Item 7	0.859	0.841	1.21	1.26	0.49	0.87	0.06
Item 8	0.868	0.851	1.10	1.13	0.71	0.88	0.06

a Based on confirmatory factor analysis.

b MNSQ: mean square.

**Table 5.** Comparison of patient characteristics by eHealth literacy level^a^.

Characteristic	Low eHealth literacy (n=385)	High eHealth literacy (n=369)	*P* value
Age (years), mean (SD)	73.05 (10.59)	68.11 (11.63)	<.001
Women, n (%)	225 (58.4)	259 (70.2)	<.001
Married or living together, n (%)	280 (72.7)	282 (76.4)	.14
Educational level >13 years, n (%)	126 (33.5)	203 (56.4)	<.001
Comorbidities present, n (%)	285 (74)	277 (75.1)	.40
RLS^b^ symptoms score, mean (SD)	5.17 (2.16)	4.92 (1.95)	.10
Sleep quality (PSQI^c^), mean (SD)	11.99 (3.69)	11.85 (3.39)	.56
Daytime sleepiness (ESS^d^), mean (SD)	10.16 (5.45)	9.63 (5.37)	.19
Shared decision-making (CollaboRATE), mean (SD)	6.13 (3.04)	6.89 (3.31)	.001
Depressive symptoms (PHQ-9^e^), mean (SD)	8.32 (6.23)	7.52 (5.29)	.05

a Participants scoring 28 or below on the eHEALS were classified as having low eHealth literacy, and those scoring above 28 were categorized as having high eHealth literacy.

b RLS: restless legs syndrome.

c PSQI: Pittsburgh Sleep Quality Index.

d ESS: Epworth Sleepiness Scale.

e PHQ-9: Patient Health Questionnaire-9.

## Discussion

### Principal Findings

This is the first study that has investigated the psychometric properties of the eHEALS among patients with RLS. Our findings showed a unidimensional structure in both the CFA and the Rasch model with high fit. The unidimensionality was not affected by age or gender, as all items were perceived similarly by younger and older patients as well as by women and men. Moreover, no substantial DIF was found across age, gender, medication use, shared decision-making condition, depressive symptoms, or sleep quality. Internal consistency also proved to be good. These findings support the use of a total score and that eHEALS can be an adequate tool to evaluate competence in health information–seeking behavior in patients of different ages with various clinical and treatment conditions related to RLS.

To begin with, a single factor solution has been found for eHEALS in several studies using general populations; among others, a recent Swedish study [43]. Diviani et al [36] found a single dimension for the Italian version in a community sample. Similarly, Mialhe et al [37], who translated the instrument into Portuguese, found excellent internal consistency for 1 dimension. However, studies focusing on different long-term conditions have reported various results. For example, Lin et al [38], who used classical test theory and Rasch analysis in a population with cardiovascular disease from Iran, found a single-factor structure. On the other hand, Richtering et al [66], who used patients with moderate to severe cardiovascular disease, found a good overall model fit, ordered response thresholds, reasonable targeting, and good internal construct validity, but that eHEALS measured 2 constructs of eHealth literacy (ie, using eHealth and understanding eHealth). Bäuerle et al [41], who evaluated the German version in patients with coronary heart disease and congestive heart failure, confirmed the 2-factor structure, construct, and criterion validity, as well as measurement invariance at the scalar level for age, gender, and educational level. Finally, Brørs et al [40], who investigated the psychometric properties in Norwegian patients after a percutaneous coronary intervention for ischemic heart disease, also found a multidimensional construct. When comparing our construct validity to the earlier-mentioned studies that have validated eHEALS in various conditions, one might have in mind that aspects of importance for the ability to seek, find, understand, and appraise health information from electronic sources may differ. These differences may be based on the presence of sociodemographic factors, such as age, gender, and education, as well as pathophysiological and symptomatologic effects related to the actual condition. Recently, the Swedish Internet Foundation reported that 95% of the adult Swedish population uses the internet, of which over 80% use eHealth services for digital health care visits or specific tasks such as prescription of medicines [2]. This may indicate that our validation carried out in a Swedish context may have slightly different prerequisites. Furthermore, when comparing our data collected during the COVID-19 pandemic to other validation studies [36-40], all conducted before the pandemic, an increased use of eHealth services in Sweden was seen during that period. Due to the social restrictions, digital alternatives then largely replaced health care delivered in a traditional way. Interestingly, even considering these aspects, the construct validity of the eHEALS appears stable, as our finding of a unidimensional factor structure supports adding the scores of individual items to calculate a total score.

Second, as patients with RLS in general are older people [16], often experience comorbidities, and report poor sleep and mood disturbances [8], as well as decreased QoL [15], it is vital to investigate DIF. Importantly, our results showed no substantial DIF for any of the items across age, gender, medication use, shared decision-making condition, depressive symptoms, and sleep quality, while item 8 showed potential DIF across sleep quality. However, the value of 0.51 was marginally above the threshold, which gives a small probability that poor sleep quality will have a decisive importance for how the current question is answered. Even if the chi-square difference test indicated a significant difference between the configural and metric models for age subgroups, the model fit indices changes were all below the established thresholds for significance. Therefore, our findings indicate the psychometric properties of eHEALS to be acceptable, which implies it to be a useful tool for researchers and clinicians to measure digital skills, informational needs, or self-care behaviors among patients with RLS. This has not been done in an RLS context, but digital skills that are deemed as important in general populations involve active information seeking, information use or sharing, and 2-way interactive communication [67]. Personal and socioeconomic factors, cultural factors, attitudes toward the internet, as well as health status have also proven to be of importance for eHealth literacy. Moreover, improved health literacy has been associated with increased health interest, promotion of health behaviors, and increased use of shared decision-making [68]. Positive relationships have also been found between eHealth literacy and various health care processes [67]. When we compared patient characteristics between low and high health literacy groups, we found that participants with higher literacy were significantly younger, more likely to be women, and more often had higher education levels. However, no significant differences were observed in civil status or comorbidity burden. Although RLS symptom severity, sleep quality, and daytime sleepiness were similar between groups, those with higher literacy reported significantly greater involvement in shared decision-making. To foster digital behaviors for care in patients with RLS without knowledge of their eHealth literacy (ie, to seek, find, understand, and appraise health information) might be difficult, especially since those with more pronounced RLS symptoms might experience significant sleep disturbances (ie, more light sleep, less deep sleep, and longer periods of wakefulness) [17], causing decreased cognitive ability [8,13]. However, using digital technology in an optimal way can be a key to developing health skills [69], which in turn can facilitate the mastery of RLS symptoms.

Fitzpatrick [69] stresses that digital tools such as eHEALS can facilitate patient education and self-care and provide empowerment possibilities. However, there are both facilitators and barriers for the implementation of digital tools for older people, which has been proven in other long-term conditions. Factors that have been found to act as both facilitators and barriers involve demographic, social, and socioeconomic factors, as well as health-related, dispositional, and technology-related factors [70]. On the other hand, facilitators often concern active engagement of the end users in the design and implementation of an eHealth program, support for overcoming concerns, privacy, and enhancing self-efficacy in the use of technology, and integration of the actual program across health services to accommodate the multimorbidity [71]. Implementing digital technology into the available RLS care can, as shown in other patient groups [68,69], probably lead to a transformation of health care delivery. Specifically, this may improve treatment options and communication among providers and patients [69], which in turn may give older patients with RLS improved involvement in their self-care as well as in RLS-related clinical decision-making [25]. However, studies on general populations have found that barriers often relate to a lack of self-efficacy, knowledge, support, functionality, and information provision about the benefits of eHealth [71]. Challenges and limitations associated with digital health literacy often include issues related to access, reliability, and privacy [69]. Therefore, the earlier-described aspects need to be explored in an RLS context using various designs and instrumentation.

Shifting the focus toward RLS care delivered through the internet, which patients today describe as a need [13], involves equipping them with digital skills to explore information to optimize their understanding of their disease and its pharmacological treatments and encouraging them to take an active role in managing their condition. However, obstacles when accessing digital health solutions concern technology literacy issues, affordability, the time burden to participate, and a perceived risk of losing in-person contact [72,73]. Studies have determined that involvement of user perspectives about what makes the best digital solution for those living with a chronic condition varies, but there is a strong conviction that tools providing feelings of reassurance increase the ability to manage their condition [72]. Several studies, not performed on patients with RLS, highlight the need for co-designing digital health interventions [72,73], as this is also particularly beneficial for providing more equitable access [72]. Guidelines for RLS treatment provide information regarding pharmacological treatment [19]. Nonpharmacological self-care interventions for RLS exist and could be assessed [74]. However, according to a meta-analysis by Harris et al [20], these are seldom used and need more evidence. Patients with RLS face several barriers to fulfill basic human needs [13] and may be perceived to be more sensitive to treatment and therefore have a greater need for easily accessible and relevant medical information than other patients [75]. A recent qualitative study [76] found that accessible information through the internet could increase motivation to perform RLS-related self-care actions. Knowledge describing self-care treatment may therefore need to be made available via digital channels to clarify self-care as a potential complement to medical treatment. Tailored patient-centered digital health care interventions, informed by the eHEALS, should be designed to promote digital health literacy at the individual and organizational level [74] to provide patients with RLS with user-friendly eHealth solutions. In this way, digital health can thus both empower and motivate various parts of RLS treatment.

### Limitations

It is important to consider some methodological aspects. Even if the sample is relatively large, the predominance of female and older retired patients may have influenced response patterns of the survey and the eHEALS as well. However, RLS is more common among women and older people [16], so our sample can be assumed to reflect the age and gender aspects of a clinical sample. The data collection was conducted via the nationwide Swedish RLS patient organization using a cross-sectional design. This, unfortunately, created a major limitation, as it limited the ability to perform test-retest analysis and explore changes in relation to different treatment interventions. Moreover, all assessments for DIF were self-reported, which might create recall bias. Future RLS studies should use a prospective design with repeated measurements to enable test-retest. They should also assess reliability and competence in health information–seeking behaviors (ie, predictive validity) in relation to self-care activities among patients with RLS.

### Conclusions

This study showed promising psychometric properties for the eHEALS among patients with RLS. The instrument operated equivalently for men and women of different ages with various clinical and treatment conditions related to RLS. Accordingly, health care professionals can use eHEALS as a psychometrically sound tool to explore the digital health literacy level among patients with RLS.

## Supplementary material

10.2196/68474 Multimedia Appendix 1 Differential item functioning analysis of the eHealth Literacy Scale across gender, age, medication use, shared decision-making status, depression status, and sleep quality status.

## Abbreviations

CFA: confirmatory factor analysis
CFI: comparative fit index
DIF: differential item functioning
eHEALS: eHealth Literacy Scale
ESS: Epworth Sleepiness scale
ICBT: internet-delivered cognitive behavioral therapy
MNSQ: infit and outfit mean square
PHQ-9: Patient Health Questionnaire-9
PSQI: Pittsburgh Sleep Quality index
QoL: quality of life
RLS: restless legs syndrome
RLS-6: Restless Legs Syndrome-6 scale
RMSEA: root mean square error of approximation
SRMR: standardized root mean square residual
TLI: Tucker-Lewis index
