# Supplementary material for: Validation of the eHealth Literacy Scale Instrument in a Restless Legs Syndrome Population: Classical Test Theory and Rasch Analysis Study
Source: J Med Internet Res. 2025 Sep 10;27:e68474. doi: 10.2196/68474 (PMC12422746; doi:10.2196/68474)
Supplement: Multimedia Appendix 1 [file jmir-v27-e68474-s001.docx]

| **DIF across gender ^abc^** | **DIF across**  **age^bd^** | **DIF**  **across**  **medi-**  **cation use ^be^** | **DIF**  **across**  **medi-**  **cation use ^bf^** | **DIF**  **across shared decision status ^bg^** | **DIF**  **across de-pression status ^bh^** | **DIF across sleep quality status ^bi^** |
| --- | --- | --- | --- | --- | --- | --- |
| -0.03 | -0.32 | 0.03 | 0.22 | -0.26 | 0 | -0.28 |
| 0 | -0.14 | 0.22 | 0.27 | -0.07 | -0.02 | -0.19 |
| 0.02 | 0 | 0.42 | 0.49 | 0 | -0.21 | 0.05 |
| 0 | 0.54 | 0.04 | -0.27 | 0 | 0.17 | -0.10 |
| 0 | 0.24 | -0.010 | -0.47 | 0.18 | 0.11 | -0.33 |
| -0.06 | -0.09 | -0.82 | -0.64 | 0.17 | 0 | 0.20 |
| 0.13 | -0.10 | 0.03 | 0.27 | -0.20 | -0.07 | 0.11 |
| -0.11 | -0.06 | 0.13 | 0.06 | 0 | 0 | 0.51 |

^a^ DIF, differential item functioning; MnSq, mean square error

^b^ DIF contrast > 0.5 indicates substantial DIF.

^c^ DIF contrast across gender=Difficulty for females-Difficulty for males.

^d^ DIF contrast across age categories = Difficulty for patients with older than 70.79 years old-Difficulty for patients with equal and younger than 70.79 years old.

^e^ DIF contrast across medication use = Difficulty for patients without medication use-Difficulty for patients with monotherapy.

^f^ DIF contrast across medication use = Difficulty for patients without monotherapy -Difficulty for patients with polytherapy.

^g^ DIF contrast across CollaboRATE total score: Difficulty for patients with SDM i.e., >6 - Difficulty for patients without SDM i.e., ≤6.

^h^ DIF contrast across PHQ9 total score: Difficulty for patients with mild depressive symptoms i.e., >10 - Difficulty for patients without depressive symptoms vs mild depressive symptoms, ≤10.

^i^ DIF contrast across PSQI total score: Difficulty for patients with sleep problems >5 - Difficulty for patients without sleep problems ≤ 5 MnSq = mean square error.
